# SupCAM: Chromosome cluster types identification using supervised contrastive learning with category-variant augmentation and self-margin loss

**DOI:** 10.3389/fgene.2023.1109269

**Published:** 2023-02-15

**Authors:** Chunlong Luo, Yang Wu, Yi Zhao

**Affiliations:** ^1^ Research Center for Ubiquitous Computing Systems, Institute of Computing Technology, Chinese Academy of Sciences, Beijing, China; ^2^ University of Chinese Academy of Sciences, Beijing, China

**Keywords:** supervised contrastive learning, category-variant data augmentation, angular margin loss, chromosome cluster types identification, karyotyping

## Abstract

Chromosome segmentation is a crucial analyzing task in karyotyping, a technique used in experiments to discover chromosomal abnormalities. Chromosomes often touch and occlude with each other in images, forming various chromosome clusters. The majority of chromosome segmentation methods only work on a single type of chromosome cluster. Therefore, the pre-task of chromosome segmentation, the identification of chromosome cluster types, requires more focus. Unfortunately, the previous method used for this task is limited by the small-scale chromosome cluster dataset, ChrCluster, and needs the help of large-scale natural image datasets, such as ImageNet. We realized that semantic differences between chromosomes and natural objects should not be ignored, and thus developed a novel two-step method called SupCAM, which could avoid overfitting only using ChrCluster and achieve a better performance. In the first step, we pre-trained the backbone network on ChrCluster following the supervised contrastive learning framework. We introduced two improvements to the model. One is called the category-variant image composition method, which augments samples by synthesizing valid images and proper labels. The other introduces angular margin into large-scale instance contrastive loss, namely *self*-margin loss, to increase the intraclass consistency and decrease interclass similarity. In the second step, we fine-tuned the network and obtained the final classification model. We validated the effectiveness of modules through massive ablation studies. Finally, SupCAM achieved an accuracy of 94.99% with the ChrCluster dataset, which outperformed the method used previously for this task. In summary, SupCAM significantly supports the chromosome cluster type identification task to achieve better automatic chromosome segmentation.

## 1 Introduction

Karyotyping is an essential cytogenetic experiment technique that aims to find numerical and structural abnormalities of chromosomes. Normally, human tissue cells have 23 pairs of chromosomes, including autosomes and sex chromosomes. These chromosomes are stained using Giemsa staining techniques and then photographed using advanced microscope cameras to generate metaphase images. The karyotyping analysis usually requires the segmentation of chromosome instances from metaphase images. Owing to the inefficiency and high cost of manual analysis, researchers have presented many automatic algorithms to ease the burden.

Most existing studies focus on the chromosome segmentation task but ignore its pre-task, chromosome cluster types identification. As non-rigid chromosomes float in an oil droplet when photographed, it is usual that touching and severely overlapping chromosomes occur in metaphase images, namely chromosome clusters. However, using classical geometric connectivity techniques, it is easy to obtain individual instances or clusters from a metaphase image. Most existing chromosome segmentation studies only dive into a specific type of chromosome cluster. To segment touching clusters, Arora (2019) and Yilmaz et al. (2018) present algorithms that make full use of the geometric characteristics between touching areas. To segment overlapping chromosome clusters, Hu et al. (2017) tries to design a new customized neural network for better performance. To segment touching-overlapping clusters, Minaee et al. (2014) dives into the geometric features of this type of cluster and proposes a geometric-based method. Alternatively, Lin et al. (2020) chooses to improve the state-of-the-art deep-learning model to tackle this issue. Nevertheless, if we can automatically identify the type of chromosome cluster first and then input it to the above segmentation methods, we can automatically segment chromosomes directly from metaphase images.

**FIGURE 1:**
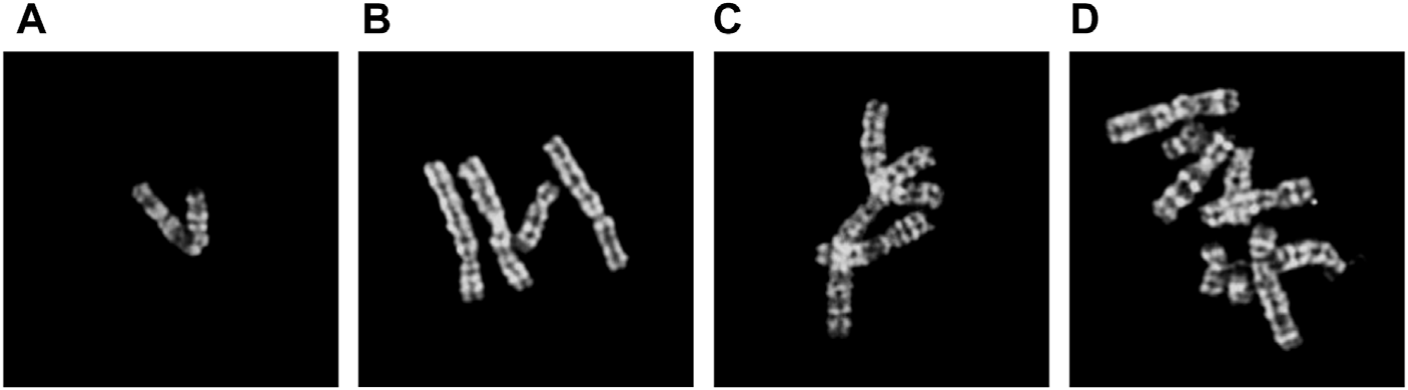
Examples of different types of chromosome clusters include **(A)** instance, **(B)** touching, **(C)** overlapping, and **(D)** touching-overlapping.

In 2021, Lin et al. (2021) proposed the chromosome cluster type identification task. In this work, 6,592 chromosome clusters were obtained from the hospital, and they created and made available the first chromosome cluster dataset (ChrCluster for simplicity). All samples are manually annotated into four categories: instance, overlapping, touching, and touching-overlapping, as shown in Figure 1. Finally, they propose a classification model as the benchmark of the ChrCluster dataset. To avoid overfitting on the small-scale ChrCluster dataset, Lin et al. (2021) takes Instagram weakly supervised learning pretrained weights [Mahajan et al. (2018)] and the customized ResNeXt [Xie et al. (2017)] classification model to achieve an accuracy of 94.09%.

However, chromosome cluster images are gray images and only contain specific domain objects, which results in different distributions between the ChrCluster dataset and the ImageNet/Instagram dataset. Therefore, pre-training the model with the ImageNet or Instagram dataset is not the ideal option. Given this point, we attempt to pre-train domain-friendly weights only using the ChrCluster dataset for better downstream task performance.

Self-supervised contrastive learning [Wu et al. (2018); van den Oord et al. (2018); Hénaff (2020); Chen T. et al. (2020a); He et al. (2020); Chen X. et al. (2020b)] is an unsupervised learning mechanism that aims to pre-train representative features (output of specific weights) that can be transferred to downstream tasks by fine-tuning. They achieve contrastive learning through a Siamese network structure. Large-scale instance contrastive loss, such as InfoNCE, is used to attract the positive pairs and repulse the negative pairs. Specifically, they regard the different augmentation views of the same instance as positive pairs and views from different instances as negative pairs. Finally, pre-trained weights are transferred to downstream tasks, such as classification, detection, and segmentation. Supervised contrastive learning methods [Khosla et al. (2020); Cui et al. (2021); Kang et al. (2021)] are further proposed to achieve better performance on the downstream classification task. They add label information into self-supervised contrastive learning. With the help of label information, not only embeddings from the different views of the same instance should be gathered together but also embeddings of instances from the same class should be pulled close, which will result in many positives for each embedding as opposed to a single positive in self-supervised contrastive learning. Given this way, we can utilize the supervised contrastive learning framework to pre-train domain-friendly features that can capture more similarity among intraclass. However, both contrastive learning methods train the model using instance contrastive loss like the SupCon loss [Khosla et al. (2020)], which means that they are non-parametric and do not have a final FC layer as a classifier. As a result, fine-tuning at the downstream chromosome cluster identification task is essential.

For both self-supervised and supervised contrastive learning, category-invariant data augmentation approaches are essential. SimCLR [Chen T. et al. (2020a)] has systematically proved the importance of category-invariant data augmentation (*RandomResizedCrop*, *RandomColorJittering*, and *GaussianBlur*) for self-supervised contrastive learning. However, stronger category-variant augmentation techniques [Zhang et al. (2018); Yun et al. (2019)] are ignored due to the lack of label information. Supervised contrastive learning methods have added label information, but the instance contrastive loss they use is not yet able to adapt to continuous labels generated by previous category-variant augmentation methods. Therefore, we introduce a category-variant image composition method with discrete targets for our proposed supervised contrastive learning method, which can further enrich the visual schemas of the ChrCluster dataset and achieve better performance.

In addition, large-scale instance contrastive loss is important for supervised contrastive learning. It is obvious that the inner product of normalized embeddings in both InfoNCE [Chen T. et al. (2020a)] and SupCon [Khosla et al. (2020)] is equal to the cosine similarity operation. The angular between two embeddings is the only variable in the loss. Thus, adding an angular margin can achieve better intraclass compactness and interclass discrimination. For example, previous angular margin-based losses [Liu et al. (2016); Liu et al. (2017); Wang et al. (2018a); Wang et al. (2018b); Deng et al. (2019)] encourage sharper feature distribution and better discriminating performance by adding various angular margins between instance features and class weights. Among them, the Additive Angular Margin loss [Deng et al. (2019)] performs best. Given this way, we can design a new large-scale instance contrastive loss using additive angular margin to enhance the semantic discrimination capability of pre-trained features.

To sum up, inspired by the supervised contrastive learning method SupCon [Khosla et al. (2020)], we propose the two-step SupCAM approach to identify the various chromosome cluster types. In the first pretraining step, considering that the MoCo [He et al. (2020)] style network can save more storage space by the memory queue, we take MoCo as feature extractor to encode images. To learn category-related features, we take SupCon loss to maximize the consistency across all views of all samples in the same class rather than only that of the various views of the same sample. Additionally, we provide a category-variant image composition method to augment chromosome cluster images, which combines two randomly chosen images and assigns a new discrete label in accordance with the rule to create a new valid sample. We also import an angular margin into different embeddings of the instance contrastive loss to bring embeddings from the same class closer together. Owing to the poor synchronization between the query and the old keys, a straightforward extension that simply adds angular margins to all positive pairs may fall short of achieving model convergency. Therefore, we only import an angular margin between the different views of the same sample, known as *self*-margin loss, which is the first attempt to enforce more compact embeddings using large-scale instance contrastive loss with angular margin. In the second step, we fine-tune the final classification model based on the pre-trained backbone from the first step. We prove the effectiveness of our methods by fine-tuning multiple classical classification networks, such as ResNet and its variants. Overall, our main contributions in this paper can be summarized as follows:

• We solve chromosome cluster identification through a two-step method, named SupCAM, that pre-trains the backbone in a supervised contrastive learning manner and fine-tunes classification models. In this way, SupCAM obtains more representative features to avoid overfitting and domain-friendly pre-trained weights as a better alternative to ImageNet pre-trained weights.
• We propose a category-variant image composition method that will reassign the category according to the overlapping area of the chromosome clusters.
• We import angular margin into instance contrastive-based loss, named *self*-margin loss. The *self*-margin loss will enforce higher intraclass compactness and interclass discrepancy of the model.
• We prove the efficiency of our contributions through the public chromosome cluster types dataset, ChrCluster. We also achieve 94.99% accuracy, which is higher than the 94.09% accuracy proposed by Lin et al. (2021).

## 2 Methods

We will go into more depth about the suggested method in this section. In the section entitled ‘Two-Step Framework’ 2.1, we fully detail the SupCAM pre-training and fine-tuning steps and emphasize the significance of the new loss function and novel data augmentation method. The details of the new category-variant image composition approach, including the composing algorithm and principles of label assigning, will thereafter be covered in the section entitled ‘Category-Variant Image Composition’ 2.2. In the section entitled ‘*Self*-margin loss’ 2.3, we will deduce new *self*-margin loss through merging label information and angular margin step by step.

### 2.1 Two-step framework

**FIGURE 2:**
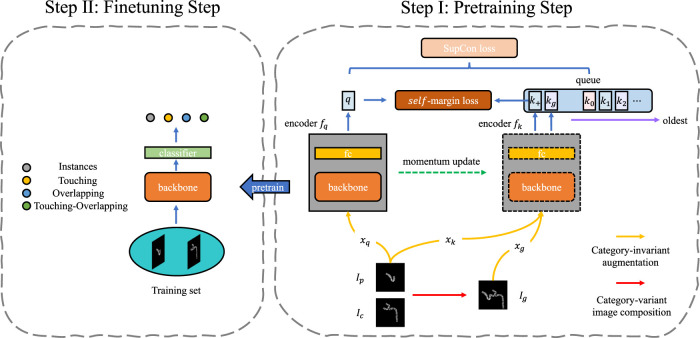
The framework of SupCAM. The pretraining step shown is based on MoCo [He et al. (2020)] structure but imports two modifications, namely category-variant image composition and *self*-margin loss. The fine-tuning step will initialize the backbone using weights pre-trained in the first step and finally reports identification results.

In this study, we present a two-step method called SupCAM that consists of the pre-training and fine-tuning steps, as shown in Figure 2, to tackle the chromosome cluster types classification problem. We pre-trained our backbone using the supervised contrastive learning framework in the first stage. In the second step, we extracted representative features through a pre-trained backbone and fine-tuned a few traditional classification models for final identification.

#### 2.1.1 Pre-training step

In the pre-training step, we took the MoCo as the basic architecture in this work, but it is free to be replaced with other self-supervised contrastive learning models. As shown in Figure 2, SupCAM owns query encoder *f*_*q*_, and key encoder *f*_*k*_. *f*_*q*_ was trained in an end-to-end manner but *f*_*k*_ was implemented as a momentum-based moving average of *f*_*q*_. We also inherited the dynamically updated queue but ignored the projection head used in the MoCo.

To gain multiple views of the sampled images during training, we first used category-invariant and category-variant augmentation approaches. Specifically, we randomly sampled primary image *I*_*p*_ and candidate image *I*_*c*_. The primary images were augmented by the category-invariant augmentation methods as usual, resulting in two views with the same class, denoted as *x*_*q*_ and *x*_*k*_. The *I*_*c*_ was first augmented using a category-variant image composition method, which combines with the *I*_*p*_ to create a new image, called generated image *I*_*g*_. A new class label was assigned according to the look-up table. Then, the same category-invariant augmentation modules were applied on the *I*_*g*_, leading to the *x*_*g*_. We will further describe the details of the category-variant image composition method in the category-variant image composition Section 2.2. Afterward, as shown in Figure 2, through the query encoder *f*_*q*_ and key encoder *f*_*k*_, augmented samples were mapped to a tuple of representation vectors:

$$\left\{q,k_{+},k_{g}\right\}=\left\{f_{q}\left(x_{q}\right),f_{k}\left(x_{k}\right),f_{k}\left(x_{g}\right)\right\}$$ (1)

where key encoder *f*_*k*_ encodes both *x*_*k*_ and *x*_*g*_ to embeddings *k*_+_ and *k*_*g*_. (*q*, *k*_+_) is the intrinsic positive pair as it comes from the same image, but *k*_*g*_ is positive or negative depending on whether *I*_*g*_ has the same class label with *I*_*p*_. Besides, *k*_+_ and *k*_*g*_ are used to update the memory queue in a first input first output (FIFO) manner. Benefiting from the slowly progressing key encoder and progressively replaced queue, representations in the queue can remain as consistent as possible with the latest *q*, which helps the contrastive model converge.

Inspired by the excellent performance of angular margin loss [Liu et al. (2017); Wang H. et al. (2018b); Deng et al. (2019)], we present *self*-margin loss in this study for better discriminative power of the pre-trained backbone. Specifically, our final loss consisted of the SupCon loss and *self*-margin loss. For each query *q*, a set of encoded keys {*k*_0_, *k*_1_, *k*_2_, … } and *k*_+_ and *k*_*g*_ were used to compute SupCon loss. Meanwhile, as *k*_+_ was not only the newest key compared with other keys in the memory queue but also had the same class as *q*, we only applied an additional angular margin between *q* and *k*_+_. In this way, we achieved better performance while keeping the training process stable. In Section 2.3, the analysis of the *self*-margin loss will be shown in detail.

#### 2.1.2 Fine-tuning step

All results shown in the section entitled ‘Experimental results and discussion’ 3 are from the fine-tuned classification model. As shown in Figure 2, in the fine-tuning step, we reused the pre-trained backbone network and attached a fully connected layer, a four-classes linear classifier, on top of it as our chromosome cluster types identification model. After loading the pre-trained weights of the backbone network and randomly initializing the fully connected layer, we trained the model on the training set for several epochs. In the end, we evaluated the SupCAM model on the test set for the module’s effectiveness and final performance. The details of the classification model and training process will be described in the section entitled ‘Implementation Details’ 3.3.

### 2.2 Category-variant image composition

In this section, we will introduce a category-variant image composition algorithm as a strong data augmentation policy in SupCAM. Traditional category-invariant data augmentation methods dominate self-supervised and supervised contrastive learning methods. However, stronger category-variant data augmentation methods, such as *Mixup* [Zhang et al. (2018)] and *CutMix* [Yun et al. (2019)], are ignored because they do not satisfy the discrete targets requirements of large-scale instance contrastive loss. Thus, we propose a category-variant image composition algorithm to synthesize new chromosome cluster samples with discrete labels for enriching visual schemas.

#### 2.2.1 Algorithm

Let (*I*_*p*_, *y*_*p*_) and (*I*_*c*_, *y*_*c*_) denote primary and candidate samples, respectively, where $\left\{I_{p},I_{c}\right\}\in R^{W\times H\times C}$ . The goal of category-variant image composition is to generate a new training sample (*I*_*g*_, *y*_*g*_) by combining primary and candidate samples. We defined the composing process as:

$$\begin{array}{cc} & I_{g}=\lambda W\left(T_{p},I_{p}\right)⊕\left(1-\lambda \right)W\left(T_{c},I_{c}\right)\\ & y_{g}=L\left(y_{p},y_{c}\right) \end{array}$$ (2)

where W represents affine function, *T*_*p*_, *T*_*c*_ are the transformation matrix of primary and candidate images, and *λ* is the combination ratio. Besides, ⊕ is complex combination operation and L means look-up table operation, which will be described in the look-up table Section 2.2.2

Algorithm 1 Category-Variant Image Composition.

**Input:** primary sample (*I*_*p*_, *y*_*p*_), candidate sample (*I*_*c*_, *y*_*c*_), upper limit of sampling number *N*, pixel intersection *P*_∩_
**Output:** generated sample (*I*_*g*_, *y*_*g*_)
1: Initialize *y*_*g*_ is uncertainty and sampling count *n* = 0
2: *W*, *H* = *Size*(*I*_*p*_)
3: Binary mask of *I*_*p*_ and *I*_*c*_:
$$M_{p}=I_{\left[I_{p}\neq 0\right]}I_{p};M_{c}=I_{\left[I_{c}\neq 0\right]}I_{c}$$
4: Bounding box of chromosome cluster in *M*_*p*_ and *M*_*c*_:
$$B_{i\in \left\{p,c\right\}}=\left(\underset{x}{\min}M_{i},\underset{x}{\max}M_{i},\underset{y}{\min}M_{i},\underset{y}{\max}M_{i}\right)$$
5: Shift range of image *I*_*p*_ and *I*_*c*_:
$$R_{i^{x}|i\in \left\{p,c\right\}}=\left[\min\left(W,W_{B_{p}}+W_{B_{c}}\right)-W_{B_{i}}\right]/2$$
$$R_{i^{y}|i\in \left\{p,c\right\}}=\left[\min\left(H,H_{B_{p}}+H_{B_{c}}\right)-H_{B_{i}}\right]/2$$
6: **while** *y*_*g*_ is uncertainty and *n* < *N* **do**
7: Shift bias are uniformly sampled according to:
$$S_{i^{x}|i\in \left\{p,c\right\}}=U\left(-R_{i^{x}},R_{i^{x}}\right)$$
$$S_{i^{y}|i\in \left\{p,c\right\}}=U\left(-R_{i^{y}},R_{i^{y}}\right)$$
8: Warp the images using transformation matrix *T*_*p*_ and *T*_*c*_: $T_{i\in \left\{p,c\right\}}=\left[\begin{array}{ccc} 1 & 0 & S_{i^{x}}\\ 0 & 1 & S_{i^{y}} \end{array}\right]$ , ${\hat{I}}_{i\in \left\{p,c\right\}}=W\left(T_{i},I_{i}\right)$ .
9: Generate *I*_*g*_ according to the warped images through combination operation ⊕:
$$I_{g}^{i,j}=\left\{\begin{array}{ccc} {\hat{I}}_{p}^{i,j} & , & \text{if}\;{\hat{I}}_{p}^{i,j}>0,{\hat{I}}_{c}^{i,j}=0\\ {\hat{I}}_{c}^{i,j} & , & \text{if}\;{\hat{I}}_{p}^{i,j}=0,{\hat{I}}_{c}^{i,j}>0\\ 0.5{\hat{I}}_{p}^{i,j}+0.5{\hat{I}}_{c}^{i,j} & , & \text{if}\;{\hat{I}}_{p}^{i,j}>0,{\hat{I}}_{c}^{i,j}>0\\ 0 & , & \text{Others} \end{array}\right.$$
10: Assign label by look-up table: $L\left(y_{p},y_{c},N_{{\hat{I}}_{p}^{i,j}\cap {\hat{I}}_{c}^{i,j}},P_{\cap }\right)$

 11: *n* = *n* + 1
 12: **if** *y*_*g*_ is not uncertainty **then**
 13: **return** Generated sample (*I*_*g*_, *y*_*g*_)
 14: **end if**
 15: **end while**
 16: **return** Candidate sample (*I*_*c*_, *y*_*c*_)

As shown in Algorithm 1, we first extracted the foreground-background mask of *I*_*p*_ and *I*_*c*_ through indicator function I and then obtained the bounding box of chromosome cluster area by min-max operation. The shift range along the *x*-axis and *y*-axis of two images is restricted by the size relation between the images and bounding boxes. Given the range, we uniformly sampled the shift bias and utilized them to construct transformation matrix $T\in R^{2\times 3}$ of image *I*_*p*_ and *I*_*c*_. The affine function W will generate transformed images according to the transformation matrix and origin images. To generate *I*_*g*_ and avoid unnatural artifacts, we designed a complex combination operation ⊕, which assigned linear interpolations of pixels only in the overlapping area. The foreground and background areas were assigned original pixels. Meanwhile, the label of *I*_*g*_ was achieved through the look-up table L . However, because of the uncertainty of *y*_*g*_, we sampled the shift bias multiple times for meaningful results but also imported an upper limit of sampling number *N* (normally 10 in our experiments) to balance the efficiency and effectiveness. Therefore, if we have sampled more than *N* times, candidate sample (*I*_*c*_, *y*_*c*_) will be directly output. The uncertainty of *y*_*g*_ will be detailed in the look-up table Section 2.2.2.

**FIGURE 3:**
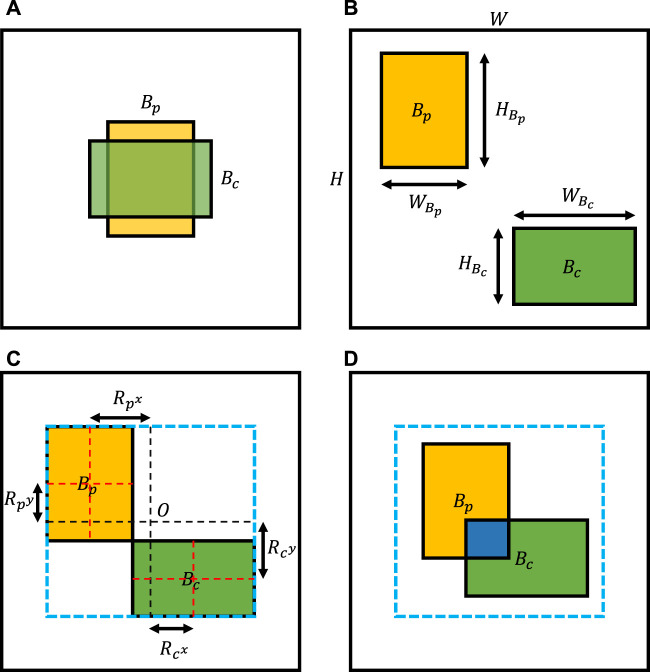
Illustration of image shift. **(A)** Common composing image without image shift. **(B)** Invalid composing image because we do not limit image shift ranges. **(C)** The image shift range determined by the maximum outer enclosing box of two bounding boxes. **(D)** Valid composing result, as we sample image shifts under a reasonable range.

Here, the importance of image shift should be clarified. Unlike *Mixup*, which conducts linear interpolations of all pixels, and *CutMix*, which replaces a random image region with a patch from another image, we need to shift the image to simulate specific properties of different types of chromosome clusters. As shown in Figure 3A, chromosome clusters are commonly distributed in the central region of the image, which means that we combine images directly without random shift, leading to overlapping instances dominating the generated samples. Additionally, we should set a limited range for the shift bias. On the one hand, unlimited shifting may lead to the loss of characteristic areas, such as overlapping or touching regions. On the other hand, as shown in the invalid image illustrated in Figure 3B, most composing results may show as two individual chromosome clusters, which do not satisfy any definition of chromosome cluster types proposed by Lin et al. (2021). To determine the range of shift bias, we simplified the irregular concave polygons of chromosome clusters to rectangles of bounding boxes. Then, two bounding boxes could uniquely confirm a maximum outer enclosing box as the border of shift bias, like Figure 3C. In this way, we are much more likely to be able to generate chromosome clusters that satisfy the definition, as shown in Figure 3D.

#### 2.2.2 Look-up table

**FIGURE 4:**
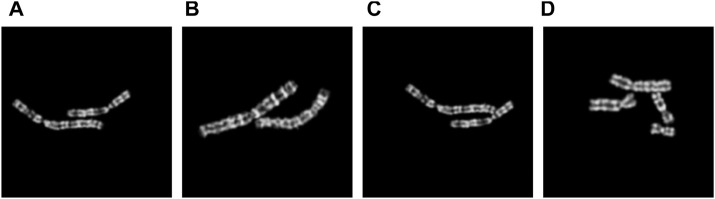
Examples of composed images with different number *N* of overlapping pixels. **(A)** *N* = 3; **(B)** *N* = 30; **(C)** *N* = 84; **(D)** *N* = 150.

In this section, we will clarify the process of assigning the correct class label to each generated sample, namely the look-up table. Considering the image composition processing and the chromosome cluster definition, the generated image will not belong to the instance category in the first place. Besides, according to Lin et al. (2021), the crucial difference between overlapping and touching chromosome clusters is whether any connectivity between two chromosome instances entails pixels intersection. However, as shown in Figure 4, it is counterintuitive if we consider these results as overlapping cases but only a few pixel intersections distribute in the pixel connectivity region. Given this point, before assigning four chromosome cluster types and an uncertainty tag, we first need to set a pixel intersection threshold *P*_∩_ greater than zero to decide whether generated image *I*_*g*_ is touching case $\left(N_{{\hat{I}}_{p}^{i,j}\cap {\hat{I}}_{c}^{i,j}}\leq P_{\cap }\right)$ or overlapping case $\left(N_{{\hat{I}}_{p}^{i,j}\cap {\hat{I}}_{c}^{i,j}}>P_{\cap }\right)$ .

**FIGURE 5:**
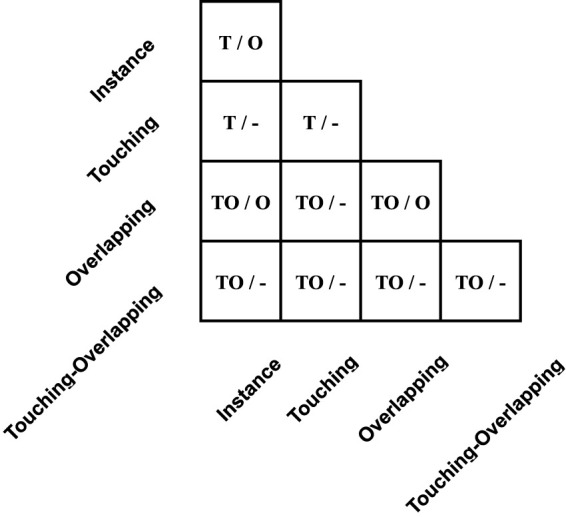
Detail of *y*_*g*_ in the *middle*-Table. The left signs in each cell represent assigned labels when the number of pixel intersections of warped *I*_*p*_ and *I*_*c*_ is below or equal to the pixel intersection threshold *P*_∩_, and the right side signs represent assigned labels when pixel intersections are larger than the threshold. Furthermore, mark ’T’, ’O’, ’TO’, and ’-’ represent touching, overlapping, touching-overlapping, and uncertainty tags, respectively.

The table in Figure 5 shows the guidance for assigning a cluster type to generated images *I*_*g*_, and for simplicity, we call it *middle*-Table. Original categories can pair into 20 possible touching and overlapping cases. As listed in *middle*-Table, the left of each cell is the candidate cluster types of touching cases, and the right is the candidate cluster types of overlapping cases. Specifically, for touching cases, their class type depends on whether touching or overlapping clusters exist in original sample pairs. In other words, only if overlapping clusters exist in original sample pairs can composed touching cases be tagged as a touching-overlapping type, such as an overlapping-instance pair. Otherwise, *y*_*g*_ should assign the touching type, such as the instance-instance pair and the touching-instance pair.

As for overlapping cases, most of the uncertainty of label *y*_*g*_ happens in this case that the number of pixel intersections beyond pixel intersection threshold *P*_∩_. Strictly speaking, except for overlapping cases of instance-instance pair, all overlapping cases should be assigned the uncertainty tag as we cannot be sure about the number of touching and overlapping regions, such as in the *light*-Table described in the section entitled Category-Variant Image Composition 3.5.3. For example, given a touching-instance pair, we can assign the touching-overlapping type or the overlapping type according to the size and position of overlapping areas between two chromosome clusters. However, we should emphasize the overlapping-instance pair and the overlapping-overlapping pair. Although two pairs can be assigned the touching-overlapping type or the overlapping type, we hypothesize that when these pairs are overlapping cases, they are unlikely to have touching areas and should directly mark the overlapping type. Finally, experiment results in Table 4 support the above hypothesis.

### 2.3 *Self*-margin loss

As in the framework shown in Figure 2, we extended the InfoNCE loss to *self*-margin loss by gradually merging label information and additive angular margin.

**FIGURE 6:**
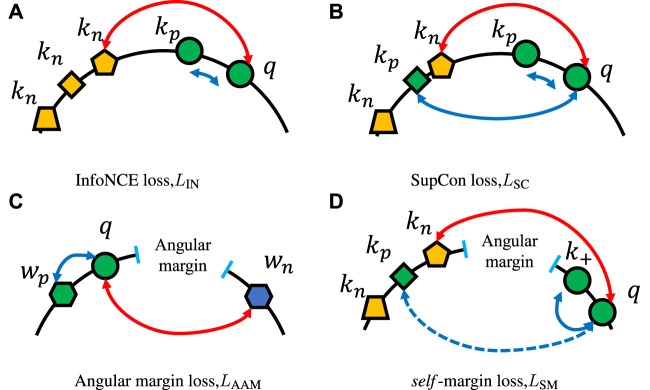
Examples of various loss functions. **(A)** InfoNCE loss, which pulls query *q* and current key *k*_*p*_ together and regards each old embedding in the memory queue as negative key *k*_*n*_, which should be pushed away. **(B)** SupCon loss, in which not only the current key but old keys that have the same class as the query should be pulled close. The example of angular margin loss in **(C)** shows how hard margin constrains parametric weights and makes the same class embeddings more compact. **(D)** Depiction of our *self*-margin loss, which only enforces the angular margin between the query and the current key and ignores other positive keys in the memory queue.

Given an encoded query $q\in R^{d}$ and a set of encoded samples {*k*_0_, *k*_1_, *k*_2_, … } stored in the memory queue, the InfoNCE loss *L*_IN_, as shown in Figure 6A considered as the following:

$$L_{\text{IN}}=-\log\frac{e^{q\cdot k_{p}/\tau }}{e^{q\cdot k_{p}/\tau }+\sum_{k_{i}\in K_{N}}e^{q\cdot k_{i}/\tau }}$$ (3)

where *k*_*p*_ is the only positive key in the memory queue that *q* matches and *K*_*N*_ represent the remaining negative key set. $\tau \in R^{+}$ is a scalar temperature parameter. In this way, *L*_IN_ is low if *q* is more in agreement with its positive key *k*_*p*_ than other negative keys, which is intuitively like a (*K*_*N*_ + 1) classes cross-entropy loss in the form.

Different from only augmented views of the same image should be considered as positives in InfoNCE loss, SupCon loss *L*_SC_ as shown in Figure 6B, imports label information and generalizes to an arbitrary number of positives as long as they belong to the same class:

$$L_{\text{SC}}=-\frac{1}{\left\|K_{P}\right\|}\underset{k_{p}\in K_{P}}{\sum }\log\frac{e^{q\cdot k_{p}/\tau }}{e^{q\cdot k_{p}/\tau }+\sum_{k_{i}\in K_{N}}e^{q\cdot k_{i}/\tau }}$$ (4)

where *K*_*P*_ is a set of positive keys that have the same class label as query *q*. The SupCon loss function can be regarded as the average of multiple times of InfoNCE loss value, as each *k*_*p*_ can be considered as the only positive key at some point. The loss encourages the encoder to pull embeddings of the same class closer, resulting in a more reasonable distribution of representations for the subsequent supervised learning task.

Now we move on to the additive angular margin loss *L*_AAM_ proposed in ArcFace [Wang F. et al. (2018a)]. As illustrated in Figure 6C, a larger angular margin, which exists between *q* and negative class weight *w*_*n*_, will enforce the same class queries *q* closer and make them easily identifiable. We suppose we have normalized weights $W\in R^{d\times \left(\|K_{N}\|+1\right)}$ of the last fully connected layer where it can be redefined as one positive class center $w_{p}\in R^{d}$ that the input matches to and remaining negative class centers $W_{N}\in R^{d\times \|K_{N}\|}$ . Additionally, we normalize its inputs *q* and ignore the bias term for simplicity. Then, the *L*_AAM_ can be reformulated as follows using our notation:

$$L_{\text{AAM}}=-\log\frac{e^{\cos\left(\theta_{q,w_{p}}+m\right)/\tau }}{e^{\cos\left(\theta_{q,w_{p}}+m\right)/\tau }+\sum_{w_{i}\in W_{N}}e^{\cos\theta_{q,w_{i}}/\tau }},$$ (5)

where $\theta_{q,w_{i}}=\arccos\left(\frac{q\cdot w_{i}}{\left\|q\|\|w_{i}\right\|}\right)$ represents the angle between *w*_*i*_ and query *q*. An additional margin penalty *m* is added on $\theta_{q,w_{p}}=\arccos\left(\frac{q\cdot w_{p}}{\left\|q\|\|w_{p}\right\|}\right)$ to enforce higher intraclass compactness and interclass discrimination.

If we set *w*_*p*_ = *k*_*p*_, *W*_*N*_ = *K*_*N*_, and *w*_*i*_ = *k*_*i*_ in *L*_AAM_, then from Eqs 4, 5 we have *self*-margin loss *L*_SM_:

$$L_{\text{SM}}=-\frac{1}{\left\|K_{P}\right\|}\underset{k_{p}\in K_{P}}{\sum }\log\frac{e^{\cos\left(\theta_{q,k_{p}}+m\right)/\tau }}{e^{\cos\left(\theta_{q,k_{p}}+m\right)/\tau }+\sum_{k_{i}\in K_{N}}e^{\cos\theta_{q,k_{i}}/\tau }}$$ (6)

However, *L*_AAM_ relies on parametric weights from the last fully connected layer. These weight vectors are the latest and are smoothly updated by end-to-end backpropagation, which results in enough synchronization between embeddings and weights. On the contrary, although a slowly evolving key encoder exists, all keys used in contrastive losses (such as *L*_IN_ and *L*_SC_) are non-parametric and rapidly changing in a FIFO manner. Given this point, positive keys are consistent enough for the contrastive-based loss but not synchronized enough for the angular margin-based loss. We cannot even make the model converge using Eq. 6.

As shown in Figure 2, the synchronization between query *q* and positive key *k*_+_ ∈ *K*_*P*_ has been guaranteed by the similar weights (moving-average key encoder *f*_*k*_ and the same batch). Therefore, in Eq. 6, for query *q*, we only hold on to the latest inherent positive key *k*_+_ and ignore the remaining positive keys, including possible *k*_*g*_. The final formulation of *self*-margin loss *L*_SM_ is:

$$L_{\text{SM}}=-\log\frac{e^{\cos\left(\theta_{q,k_{+}}+m\right)/\tau }}{e^{\cos\left(\theta_{q,k_{+}}+m\right)/\tau }+\sum_{k_{i}\in K_{N}}e^{\cos\theta_{q,k_{i}}/\tau }}$$ (7)

and illustrated in Figure 6D.

We will prove the performance of *L*_SM_ in Experiments 3.5 and compare it with some intuitive candidate methods.

## 3 Experimental results and discussion

### 3.1 Dataset

In this study, we used the dataset reported by Lin et al. (2021) to evaluate our model performance and demonstrate the effectiveness of modules. The dataset is the first clinical chromosome cluster dataset that has 6,592 samples, called ChrCluster. All samples are padded to the 224 × 224 size and manually labeled into four categories: 1,712 chromosome instance, 3,029 touching chromosomes cluster, 1,038 overlapping chromosomes cluster, and 813 touching-overlapping chromosomes cluster. In the ablation study Section 3.5, we described how we split the dataset into 3,955 training samples, 659 validation samples, and 1,978 test samples in a class-based random stratified fashion. For the final comparison in the Section entitled ‘Comparison Result’ 3.4, we followed the division principle described by Lin et al. (2021), which has 80% training data, 10% validation data, and 10% test data. To avoid leaking test set information from the pre-training step to the fine-tuning step, we pre-trained the backbone network only using the training set no matter whether the goal is an ablation study or final comparisons.

### 3.2 Evaluation metrics

To fairly evaluate the performance of SupCAM, we followed the main evaluation metrics described by Lin et al. (2021) including *accuracy*, *precision*, *sensitivity*, *specificity*, and *F1*. It is worth noticing that except for the *accuracy*, all the above-mentioned metrics were averaged in a ‘macro’ fashion. The ‘macro’ fashion will first calculate metrics for each category individually and then average the metrics across classes with equal weights.

Now, we should clarify the definition of the following four basic criteria in a multi-classification task:

• True positive(*TP*_*i*_): given a test sample that belongs to *i*-th class, if the model correctly predicts it as *i*-th class, we regard it as true positive.
• False positive(*FP*_*i*_): given a test sample that does not belong to *i*-th class, if the model incorrectly predicts it as *i*-th class, we regard it as false positive.
• False negative(*FN*_*i*_): given a test sample that belongs to *i*-th class, if the model incorrectly predicts it as other classes, we regard it as false negative.
• True negative(*TN*_*i*_): given a test sample that does not belong to *i*-th class, if the model correctly predicts it as other classes, we regard it as true negative.

Assume that *N*_*c*_ represents the number of chromosome cluster categories and *N* is the number of test set instances, then we have:

$$accuracy=\frac{1}{N}\overset{N_{c}}{\underset{i=0}{\sum }}TP_{i}$$ (8)

$$\begin{array}{cc} precision & =\frac{1}{N_{c}}\overset{N_{c}}{\underset{i=0}{\sum }}precision_{i}\\ & =\frac{1}{N_{c}}\overset{N_{c}}{\underset{i=0}{\sum }}\frac{TP_{i}}{TP_{i}+FP_{i}} \end{array}$$ (9)

$$\begin{array}{cc} sensitivity & =\frac{1}{N_{c}}\overset{N_{c}}{\underset{i=0}{\sum }}sensitivity_{i}\\ & =\frac{1}{N_{c}}\overset{N_{c}}{\underset{i=0}{\sum }}\frac{TP_{i}}{TP_{i}+FN_{i}} \end{array}$$ (10)

$$\begin{array}{cc} specificity & =\frac{1}{N_{c}}\overset{N_{c}}{\underset{i=0}{\sum }}specificity_{i}\\ & =\frac{1}{N_{c}}\overset{N_{c}}{\underset{i=0}{\sum }}\frac{TN_{i}}{TN_{i}+FP_{i}} \end{array}$$ (11)

$$F1=\frac{1}{N_{c}}\overset{N_{c}}{\underset{i=0}{\sum }}2\cdot \frac{precision_{i}\cdot sensitivity_{i}}{precision_{i}+sensitivity_{i}}$$ (12)

All above-mentioned metrics are as higher as better. We use percentages for them and keep two decimal places.

### 3.3 Implementation details

We implemented our work on the Pytorch Lightning^1^ toolbox based on the Pytorch [Paszke et al. (2019)] deep-learning library. We finished all experiments on an Ubuntu OS Server with one NVIDIA GTX Titan Xp GPU.

In the first pre-training phase, following the MoCo pipeline, we optimized the structure and some hyperparameters for the chromosome cluster type identification task. As described in the two-step framework Section 2.1, besides the conventional query *q* and key *k*_+_ used in MoCo, we additionally generated *x*_*g*_ using the category-variant image composition method and encoded it as the third embedding *k*_*g*_ through the key encoder. Limited by the size of the dataset, we reduced the embedding dimension to 128-d and the queue capacity to 1,024 accordingly. The scalar temperature *τ* used in SupCon loss and *self*-margin loss was set as 0.07. We chose 0.2 for angular margin *m* and 200 for pixel intersection hyperparameter *P*_∩_. We used SGD as our optimizer, where momentum is 0.9, and weight decay is 0.0001. We set the mini-batch size as 32 for the single GPU and trained the model for 200 epochs. Furthermore, we applied linear warm-up during the first 20 epochs until achieving the initial learning rate 0.03 and then decayed it through a cosine annealing schedule. Category-invariant image augmentation methods used in the first step included *RandomResizedCrop* and *HorizontalFlip*. The total loss is the sum of SupCon loss *L*_SC_ and *self*-margin loss *L*_SM_:

$$L=L_{\text{SC}}+L_{\text{SM}}$$ (13)

In the second fine-tuning step, for the classification model, we first loaded the corresponding pre-trained backbone module and randomly initialized the weights and bias of the final classifier. Only *RandomRotate* was employed during the training phase to reduce overfitting. We used SGD as our optimizer and had the same setting as the pre-training step. We trained the classification model for 15 epochs with a mini-batch of 16 images. Unlike the first step, the initial learning rate was set as 0.01 and decreased by 0.1 after 8 and 12 epochs individually. The loss function adopted in the fine-tuning step was cross-entropy loss enhanced by label smoothing (hyperparameter *σ* = 0.1) [Szegedy et al. (2016)].

### 3.4 Comparison result

#### 3.4.1 Overview

In this section, we report the final results following the division principle of Lin et al. (2021). Table 1 shows the comparison results between SupCAM and previous methods. On the top of Table 1, we list some representative experiment results of previous methods with different backbones, including ResNet101 [He et al. (2016)], DenseNet121 [Huang et al. (2017)], and ResNeXt101 [Xie et al. (2017)]. Specifically, ResNeXt^†^ optimizes the header of the classification model using a mixed pooling layer and multiple linear-dropout groups. Meanwhile, not only 1.28 million images from the ImageNet dataset but also approximately 940 million images from the Instagram dataset are used to pre-train backbone weights, which are loaded as initial weights of the ResNeXt^†^. Owing to above-mentioned improvements, ResNeXt^†^ proposed by Lin et al. (2021) achieves the previous state-of-the-art performance, which is 94.09 accurate and has the best results with other evaluation metrics.

**TABLE 1:** Comparison with previous methods. All experiments were conducted following the division principle in Lin et al. (2021). ResNeXt101: ResNeXt101 32 × 8d; ^†^: ResNeXt101-32 × 8d attached with a customized header network invented by Lin et al. (2021); ImageNet: 1.28 million images with 1,000-class ImageNet dataset; Instagram: 940 million public images with a ∼1500 hashtags dataset proposed by Mahajan et al. (2018).

Methods	Backbone	Pre-train dataset	Accuracy	Precision	Sensitivity	Specificity	F1
Lin et al. (2021)	ResNet101	ImageNet(1.28 M)	91.89	90.65	87.92	97.30	88.32
	DenseNet121	ImageNet(1.28 M)	87.65	85.59	81.68	95.88	82.23
	ResNeXt101	ImageNet(1.28 M)	92.27	90.79	89.10	97.42	89.36
	ResNeXt101^†^	Instagram(940 M)	94.09	93.08	92.79	98.03	**92.84**
SupCAM	ResNet101	ChrCluster(6.5K)	94.24	92.54	92.00	97.74	91.37
	DenseNet121	ChrCluster(6.5K)	94.69	92.92	92.89	97.94	92.11
	ResNeXt101	ChrCluster(6.5K)	**94.99**	**93.25**	**92.81**	**98.12**	92.26

The bold values represent that they are the best performance in this metric.

In this study, benefiting from the supervised contrastive learning framework enhanced by the category-variant image composition methods and *self*-margin loss, SupCAM achieved the best performance. Specifically, SupCAM improved the accuracy by a large margin of 2.35 under ResNet101 and 2.72 under the original ResNeXt101. Finally, although Lin et al. (2021) used an extremely large Instagram dataset, which was almost 140,000 times larger than ChrCluster, we still increased the accuracy by approximately 0.9 compared with ResNeXt^†^. Except for F1, other metrics also performed better. It is worth noting that previous methods may suffer from heavy overfitting, as shown in the result that used the DenseNet121 as the backbone network in Lin et al. (2021). As a more powerful backbone than ResNet101, DenseNet121 performed less well in all metrics. By contrast, under SupCAM, DenseNet121 successfully outperformed ResNet101, which means that SupCAM can alleviate the risk of overfitting without relying on a large dataset but using only the ChrCluster dataset. To sum up, Table 1 shows the high data utilization efficiency and robustness of the SupCAM for solving the task of chromosome cluster type identification. In addition, we evaluated the performance using pre-trained weights from ImageNet instead of random initialization in the first step of SupCAM, and as shown in Supplementary Table S1, it also outperformed the previous method, but was worse than the final SupCAM.

#### 3.4.2 Confusion matrix

**FIGURE 7:**
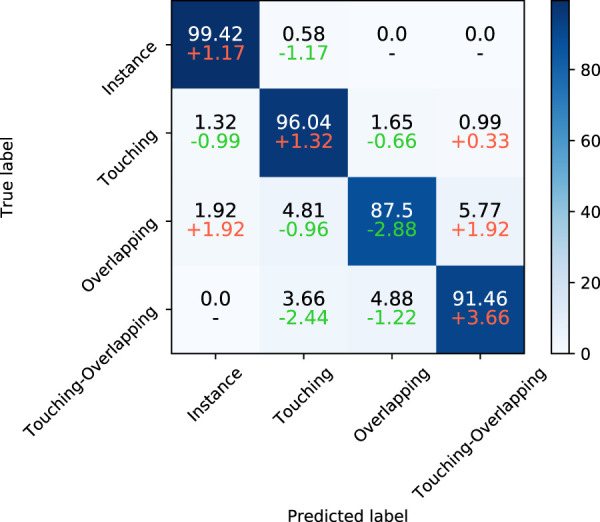
The first line of each cell is the confusion matrix of SupCAM with ResNeXt101 backbone on the test set. Besides, on the second line of each cell, we show differences between Lin et al. (2021) and our method, in which red and green represent increment or decrement of percentage, respectively.

Besides the above metrics across classes, we used a confusion matrix to further reveal the performance of the SupCAM method in each class. As shown in Figure 7, SupCAM outperformed a previous study [Lin et al. (2021)] on instance, overlapping, and touching-overlapping classes but was weak in the overlapping category. Specifically, the number of touching-overlapping clusters incorrectly predicted as touching and overlapping types were reduced simultaneously, which resulted in an increment of 3.66 in the accuracy of the touching-overlapping class. Additionally, the accuracy of the instance class and the touching class was increased to 99.42 and 96.04, respectively. It is obvious that the combination of the category-variant image composition method and *self*-margin loss can improve the performance of the identification model in most chromosome cluster categories.

**FIGURE 8:**
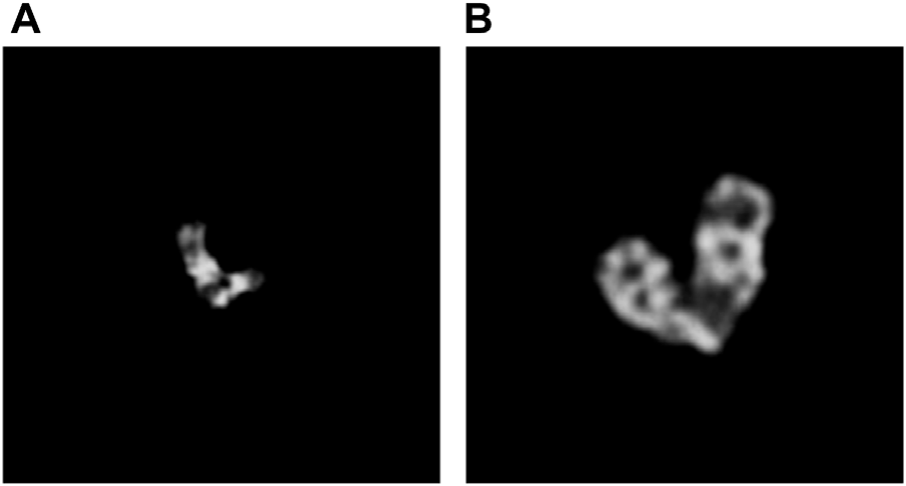
Overlapping clusters are misclassified as the instance class by SupCAM. **(A)** shows a misclassified example where the bottom of the left chromosome occludes the other one. **(B)** is another misclassified example where the bottom of the right chromosome occludes the bottom of the left one.

At the same time, to try to explain the degeneracy of SupCAM in the overlapping category, we list some false negative samples, especially those misclassified as the instance type. As illustrated by Figure 8, they are puzzling samples, and it is hard to decide whether they belong to the overlapping type at first glance. On the other side, a hard threshold of pixel intersection in the category-variant image composition method may import artificial disturbance to the label system, which adds confusion to the final prediction. Therefore, these weaknesses inspire us to propose more reasonable and natural image composition methods in the future.

### 3.5 Ablation study

#### 3.5.1 Overview

To evaluate the effectiveness of each model, we applied the ablation study at the 30% test set of the ChrCluster dataset. To avoid performance fluctuations due to the small size of the dataset, all experiments during the ablation study were repeated 10 times and we obtained the mean and standard deviation of each evaluation metric. In this way, as well as comparing the performance through the mean value, we can further justify the stableness of each method.

As shown in Table 2, we first trained the chromosome cluster types classification model from scratch as the baseline, which was 88.38 ± 0.60 accurate. Pre-training on the large ImageNet dataset further improved the accuracy to 92.65 ± 0.30. However, the above experiments suffer from larger performance fluctuation than our methods, which reminds us that a huge domain gap exists between the ImageNet and ChrCluster. Therefore, pre-training the chromosome cluster types identification model on the large ImageNet dataset is not the best choice. Finally, we proved that the key factor driving the model performance improvement is the model structure itself as SupCAM achieved the best performance among all experiments under the same fine-tuning strategies.

**TABLE 2:** Ablation study of the SupCAM model with ResNet50 on the 30% test set of the ChrCluster dataset. We repeated all experiments 10 times and report the mean and standard deviation. IN indicates that the backbone network has been pre-trained by the ImageNet dataset. CatVar, category-variant image composition method; *L*_SM_, *self*-margin loss.

IN	MoCo	SupCon	CatVar	*L*_SM_	Accuracy	Precision	Sensitivity	Specificity	F1
					88.38 ± .60	84.07 ± .82	83.94 ± .79	95.79 ± .19	82.11 ± .88
*✓*					92.65 ± .30	90.24 ± .65	89.87 ± .73	97.31 ± .10	88.79 ± .72
	*✓*				89.15 ± .34	85.31 ± .60	85.23 ± .49	95.97 ± .15	83.61 ± .53
	*✓*	*✓*			91.65 ± .32	88.08 ± .51	88.60 ± .38	96.97 ± .13	87.00 ± .47
	*✓*	*✓*	*✓*		93.24 ± .20	91.18 ± .46	90.60 ± .40	97.49 ± .09	89.75 ± .46
	*✓*	*✓*	*✓*	*✓*	**93.56** ± **.18**	**91.65** ± **.42**	**91.31** ±**.36**	**97.63** ± **.06**	**90.34** ± **.41**

The bold values represent that they are the best performance in this metric.

#### 3.5.2 Supervised contrastive learning

To verify the contribution of supervised contrastive learning to the performance, before completing the basic classification task, we imported the pre-training step, which pre-trained the backbone in a supervised contrastive manner with SupCon loss through MoCo architecture. We took the MoCo augmentation setting [Chen X. et al. (2020b)] as the initial augmentation method in this experiment. Table 2 shows that the MoCo-style supervised contrastive pre-training step increased accuracy by 3.27 points and had a F1 score 4.89 points higher than the model trained from scratch. It is notable here that the direct employment of the MoCo-style supervised contrastive pre-training step was worse than the identification model pre-trained by the ImageNet dataset, but it was more stable in some cases. In conclusion, pre-training the backbone in a supervised contrastive manner is effective but we need more specific optimizations to adapt the chromosome cluster types identification task.

#### 3.5.3 Category-variant image composition

The experiment results in Table 2 show that the category-variant image composition method improves accuracy from 91.65 ± 0.32 to 93.25 ± 0.20 and specificity from 96.97 ± 0.13 to 97.49 ± 0.09. Both the performance and stableness of this model were increased and even outperformed the model trained by the MoCo setting, which validates that the category-variant image composition method can more reasonably and effectively augment chromosome cluster data than the original MoCo augmentation setting.

To be more specific, as shown in Supplementary Figure S1, we experimented with multiple candidate pixel intersection threshold *P*_∩_, and box plots show that when the *P*_∩_ is set as 200 pixels, the model achieves the best performance in all metrics. Meanwhile, we also examined the choices of composition methods in overlapping areas, as shown in Table 3. Besides the equal weights method used in this study, we list two representative composition methods. Linear interpolation through a sampled *λ* ∼B(1,1) is widely used in Yun et al. (2019) and Zhang et al. (2018). Another straightforward idea is taking the maximum pixel value from the primary image *I*_*p*_ and the candidate image *I*_*c*_ as the final pixel in overlapping areas. Experiments show that the ‘maximum’ method is not suitable for the chromosome cluster types identification task and the ”*λ*-interpolation” method performs badly on the most important accuracy criterion, although slightly outperforms the ‘equal weights’ method on other metrics.

**TABLE 3:** Ablation study of composition methods. Equal weights mean that the overlapping area of *I*_*p*_ and *I*_*c*_ are combined half and half. *λ* shows that we sampled a *λ* from beta distribution and then applied linear interpolations in the overlapping areas of two images. The final maximum experiment represents the operation of taking the maximum pixel value in overlapping areas.

Composition method	Accuracy	Precision	Sensitivity	Specificity	F1
Equal weights	**93.56** ± **.18**	91.65 ± .42	91.31 ± .36	**97.63 ± .06**	90.34 ± .41
*λ*-interpolation [Yun et al. (2019)]	93.41 ± .21	**91.66** ± **.37**	**91.66** ± **.35**	**97.62** ± **.09**	**90.47** ± **.35**
Maximum	93.04 ± .17	89.93 ± .37	89.93 ± .36	97.44 ± .08	88.77 ± .36

The bold values represent that they are the best performance in this metric.

Furthermore, we confirmed the design of the look-up table in Table 4. As shown in the results, the *middle*-Table scheme achieved the best performance. In addition, we evaluated some extreme scenarios, such as the *heavy*-Table scheme and the *light*-Table scheme. Specifically, the *heavy*-Table scheme assigns an explicit label to each (*I*_*p*_, *I*_*c*_) pair directly no matter whether disagreements exist in overlapping cases. Suppose there is a touching-instance pair in an overlapping case, the *middle*-Table will tag them with an uncertainty label, but with a *heavy*-Table, we roughly assign the touching-overlapping category. The *light*-Table solution takes the opposite approach by not providing any valid label for overlapping cases unless they all belong to the instance type. The results in Table 4 show that the *heavy*-Table achieved an accuracy of 93.30 ± .28, which outperformed the 93.09 ± .11 accuracy of the *light*-Table scheme. Through the comparison between *light*-*middle*-*heavy* solutions, we can conclude that 1) category-variant image composition method indeed improves the performance of the cluster type identification task $\left(\mu_{\text{heavy}}^{\mathrm{Acc}}>\mu_{\text{light}}^{\mathrm{Acc}}\right)$ ; 2) we should avoid roughly assigning a label for complicated cases $\left(\mu_{\text{middle}}^{\mathrm{Acc}}>\mu_{\text{heavy}}^{\mathrm{Acc}}\right)$ ; and 3) manually composing an image and assigning a label inevitably imports unnatural counterfeits, resulting in performance fluctuation $\left(\sigma_{\text{heavy}}^{\mathrm{Acc}}>\sigma_{\text{middle}}^{\mathrm{Acc}}>\sigma_{\text{light}}^{\mathrm{Acc}}\right)$ .

**TABLE 4:** Ablation study of the look-up table. Besides the *middle*-Table, which was our final choice, we tried to extend the label assigning to the extreme, namely through the *heavy*-Table and *light*-Table schemes. The goal of the *no*-Table is to examine the effects of candidate image *I*_*c*_.

Scheme	Accuracy	Precision	Sensitivity	Specificity	F1
*middle*-Table	**93.56** ± .18	**91.65** ± .42	**91.31** ± .36	**97.63** ± .06	**90.34** ± .41
*heavy*-Table	93.30 ± .28	90.76 ± .56	90.56 ± .56	97.55 ± .11	89.52 ± .57
*light*-Table	93.09 ± .11	90.77 ± .34	90.66 ± **.29**	97.47 ± **.05**	89.50 ± **.31**
*no*-Table	93.19 ± **.10**	90.84 ± **.31**	90.71 ± .40	97.53 ± **.05**	89.56 ± .36

The bold values represent that they are the best performance in this metric.

Moreover, to clarify the effects of taking *I*_*c*_ as *I*_*g*_ as in line 16 of Algorithm 1; Table 4 shows the results from a contrast experiment we conducted, called a *no*-Table scheme, that only used existing candidate image *I*_*c*_ rather than composed images. As expected, *no*-Table achieved an accuracy of 93.19 ± .10, which was lower but more stable than that of *middle*-Table, proving the effectiveness and relative unstableness of the category-variant image composition method once more.

#### 3.5.4 SupCAM with *Self*-margin loss

As shown in Table 2, *self*-margin loss improved the accuracy from 93.24 ± .20 to 93.56 ± .18 and the precision, sensitivity, specificity, and F1 scores were also improved. Besides, it is worth noting that weights pre-trained with *self*-margin loss could further stabilize the final classification performance. Thus, we validated the effectiveness of *self*-margin loss of the first pre-training step.

It is important to find the optimal margin *m* for the chromosome cluster types identification task, and the best margin *m* observed in Table 5 was 0.2. Specifically, smaller additional angular margin penalties, such as *m* = 0.1 and *m* = 0.2, improved the performance. However, when margin penalties was large, e.g., *m* = 0.3 and *m* = 0.4, *self*-margin loss not only decreased the performance but also made the model more unstable. When the margin penalty increased to 0.5, the model could not be converged. Therefore, we conclude that although we ensure synchronization by (*q*, *k*_+_) pair, the moving-average update manner makes the model more sensitive to the large margin penalty than the model updated in an end-to-end manner, which is further described in the next paragraph.

**TABLE 5:** The table below shows SupCAM performance with different angular margin values (*m* in Eq. 7) used in the *self*-margin loss during the first pre-training step.

Angular margin	Accuracy	Precision	Sensitivity	Specificity	F1
*m* = 0.1	93.39 ± **.15**	91.10 ± **.29**	90.92 ± **.35**	97.59 ± .07	89.90 ± **.37**
*m* = 0.2	**93.56** ± .18	**91.65** ± .42	**91.31** ± .36	**97.63** ± **.06**	**90.34** ± .41
*m* = 0.3	93.21 ± .26	90.62 ± .57	90.73 ± .52	97.51 ± .10	89.46 ± .58
*m* = 0.4	91.87 ± .35	88.50 ± .42	88.42 ± .42	97.10 ± .12	87.04 ± .53
*m* = 0.5	\	\	\	\	\

The bold values represent that they are the best performance in this metric.

**FIGURE 9:**
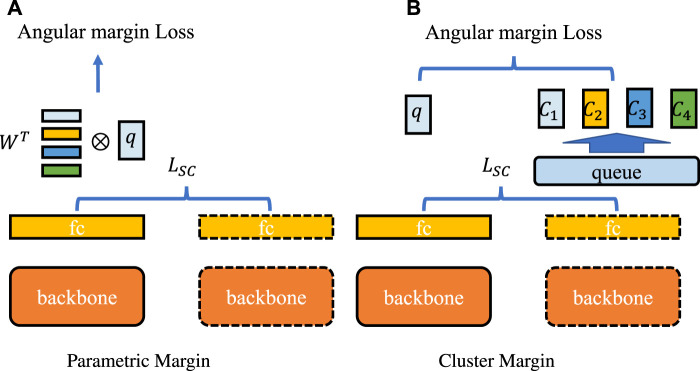
Comparison of margin-based methods. The backbone network is shared between the first and second steps, and the fully connected layer outputs queries and keys. The dashed lines indicate that all layers are updated in a moving-average manner. *L*_SC_ represents SupCon loss. **(A)** represents the “parametric margin” schema which applies angular margin loss between query embedding and additional parametric weights *W*. **(B)** is the “cluster margin” method that clusters all key embeddings into four class centers {*C*_1_, *C*_2_, *C*_3_, *C*_4_} according to class label, and applies angular margin between query embeddings and cluster centers.

Furthermore, margin-based architectures are diverse, and we justified the advantages of *self*-margin loss through the results shown in Table 6. As illustrated in Figure 9A, with ‘parametric margin’ as one of the candidate schemes, we additionally added an end-to-end updating weight $W\in R^{d\times 4}$ as classes centers after the original fully connected layer and the angular margin-based loss is applied between the parametric weights and query embedding *q*. Results proved that the ‘Parametric Margin’ scheme is not good at the chromosome cluster types identification task; however, its better stability also confirms the conclusion in the above paragraph. Another candidate scheme is ‘cluster margin’, as shown in Figure 9B. To form meaningful class centers for each query *q*, we clustered all key embeddings stored in the memory queue according to their label and renormalized the center of each cluster. Cluster centers were updated in a moving-average manner. However, the results in Table 6 confirmed what we inferred in the *self*-margin loss’ Section 2.3, i.e., that terrible synchronization leads to worse performance under the angular margin framework.

**TABLE 6:** Other candidate angular margin based scenarios and the main differences are detailed in Section 3.5.4.

Other angular margin method	Accuracy	Precision	Sensitivity	Specificity	F1
*self*-margin loss	**93.56** ± .18	**91.65** ± .42	**91.31** ± .36	**97.63** ± .06	**90.34** ± .41
Parametric margin(*m* = 0.2)	93.29 ± .20	90.88 ± .38	91.10 ± .34	97.56 ± .08	89.94 ± .35
Parametric margin(*m* = 0.3)	93.13 ± .15	91.03 ± .34	90.88 ± .32	97.49 ± .07	89.83 ± **.29**
Parametric margin(*m* = 0.4)	93.08 ± .19	90.66 ± .36	90.44 ± **.24**	97.47 ± .06	89.40 ± .32
Parametric margin(*m* = 0.5)	93.26 ± **.13**	91.42 ± **.31**	91.03 ± .29	97.56 ± **.05**	90.07 ± .30
Cluster margin	93.09 ± .18	90.57 ± .42	90.74 ± .49	97.51 ± .08	89.49 ± .47

The bold values represent that they are the best performance in this metric.

## 4 Conclusion

In this study, we proposed a two-step SupCAM method to solve the chromosome cluster types identification task. In the first step, we improved the supervised contrastive learning method through a strong category-variant image composition algorithm and *self*-margin loss. After pre-training, we further fine-tuned the classification models in the second step. The effectiveness of each module was proved by massive ablation studies. The top prediction performance suggested that SupCAM has state-of-the-art performance in the chromosome cluster identification task. All these experimental findings demonstrate that the proposed SupCAM, as a supervised contrastive learning method, can effectively extract more representative and domain-friendly weights from the small-scale ChrCluster and is a better alternative to previous ImageNet pre-trained weights as it alleviates overfitting risks, resulting in better performance. Specifically, SupCAM introduces a strong category-variant image composition method with discrete labels to generate more abundant visual schemas. Meanwhile, we designed and implemented a new stable *self*-margin loss by adding an angular margin between the different embeddings of the instance contrastive loss, resulting in higher intraclass compactness and interclass discrepancy. Although our study focuses on chromosome cluster identification, our proposed method can inspire more researchers to analyze medical images using only small-scale medical image datasets rather than large natural image datasets. In the future, we will refine image composition processing and the look-up table to achieve more stable performance. In addition, other schemes that add angular margin into instance contrastive-based loss should be further studied.

## Data Availability

Publicly available datasets were analyzed in this study. This data can be found here: [https://github.com/ChengchuangLin/ChromosomeClusterIdentification].

## Footnotes

1
https://pytorch-lightning.readthedocs.io/en/latest/

## References

[B1] Arora T. (2019). A novel approach for segmentation of human metaphase chromosome images using region based active contours. Int. Arab. J. Inf. Technol. 16, 132–137.

[B2] Chen T. Kornblith S. Norouzi M. Hinton G. E. (2020a). “A simple framework for contrastive learning of visual representations,” in Proceedings of the 37th International Conference on Machine Learning, ICML 2020, Virtual Event (PMLR), 13-18 July 2020 (Vienna, Austria: PMLR), 1597–1607. vol. 119 of Proceedings of Machine Learning Research.

[B3] Chen X. Fan H. Girshick R. B. He K. (2020b). Improved baselines with momentum contrastive learning. CoRR abs/2003.04297.

[B4] Cui J. Zhong Z. Liu S. Yu B. Jia J. (2021). “Parametric contrastive learning,” in 2021 IEEE/CVF International Conference on Computer Vision, ICCV 2021, October 10-17, 2021 (Montreal, QC, Canada: IEEE), 695–704. 10.1109/ICCV48922.2021.00075

[B5] Deng J. Guo J. Xue N. Zafeiriou S. (2019). “Arcface: Additive angular margin loss for deep face recognition,” in IEEE Conference on Computer Vision and Pattern Recognition, CVPR 2019, June 16-20, 2019 (Long Beach, CA, USA: Computer Vision Foundation/IEEE), 4690–4699. 10.1109/CVPR.2019.00482

[B6] He K. Fan H. Wu Y. Xie S. Girshick R. B. (2020). “Momentum contrast for unsupervised visual representation learning,” in 2020 IEEE/CVF Conference on Computer Vision and Pattern Recognition, CVPR 2020, June 13-19, 2020 (Seattle, WA, USA: Computer Vision Foundation/IEEE), 9726–9735. 10.1109/CVPR42600.2020.00975

[B7] He K. Zhang X. Ren S. Sun J. (2016). “Deep residual learning for image recognition,” in 2016 IEEE Conference on Computer Vision and Pattern Recognition, CVPR 2016, June 27-30, 2016 (Las Vegas, NV, USA: IEEE Computer Society), 770–778. 10.1109/CVPR.2016.90

[B8] Hénaff O. J. (2020). “Data-efficient image recognition with contrastive predictive coding,” in Proceedings of the 37th International Conference on Machine Learning, ICML 2020, Virtual Event (PMLR), vol. 119 of Proceedings of Machine Learning Research, 13-18 July 2020 (Vienna, Austria: PMLR), 4182–4192.

[B9] Hu R. L. Karnowski J. Fadely R. Pommier J. (2017). Image segmentation to distinguish between overlapping human chromosomes. CoRR abs/1712.07639.

[B10] Huang G. Liu Z. van der Maaten L. Weinberger K. Q. (2017). “Densely connected convolutional networks,” in 2017 IEEE Conference on Computer Vision and Pattern Recognition, CVPR 2017, July 21-26, 2017 (Honolulu, HI, USA: IEEE Computer Society), 2261–2269. 10.1109/CVPR.2017.243

[B11] Kang B. Li Y. Xie S. Yuan Z. Feng J. (2021). “Exploring balanced feature spaces for representation learning,” in 9th International Conference on Learning Representations, ICLR 2021, Virtual Event, May 3-7, 2021 (Austria: OpenReview.net).

[B12] Khosla P. Teterwak P. Wang C. Sarna A. Tian Y. Isola P. (2020). “Supervised contrastive learning,” in Advances in Neural Information Processing Systems 33: Annual Conference on Neural Information Processing Systems 2020, NeurIPS 2020, virtual, December 6-12, 2020.

[B13] Lin C. Zhao G. Yin A. Ding B. Guo L. Chen H. (2020). As-panet: A chromosome instance segmentation method based on improved path aggregation network architecture. J. Image Graph. 25, 2271–2280.

[B14] Lin C. Zhao G. Yin A. Yang Z. Guo L. Chen H. (2021). A novel chromosome cluster types identification method using resnext WSL model. Med. Image Anal. 69, 101943. 10.1016/j.media.2020.101943 33388457

[B15] Liu W. Wen Y. Yu Z. Li M. Raj B. Song L. (2017). “Sphereface: Deep hypersphere embedding for face recognition,” in 2017 IEEE Conference on Computer Vision and Pattern Recognition, CVPR 2017, July 21-26, 2017 (Honolulu, HI, USA: IEEE Computer Society), 6738–6746. 10.1109/CVPR.2017.713

[B16] Liu W. Wen Y. Yu Z. Yang M. (2016). “Large-margin softmax loss for convolutional neural networks,” in Proceedings of the 33nd International Conference on Machine Learning, ICML 2016, June 19-24, 2016 (New York City, NY, USA: JMLR.org), 507–516. vol. 48 of JMLR Workshop and Conference Proceedings.

[B17] Mahajan D. Girshick R. B. Ramanathan V. He K. Paluri M. Li Y. (2018). “Exploring the limits of weakly supervised pretraining,” in Computer Vision - ECCV 2018 - 15th European Conference, September 8-14, 2018 (Munich, Germany: Springer), 185–201. Proceedings, Part II. vol. 11206 of Lecture Notes in Computer Science. 10.1007/978-3-030-01216-8_12

[B18] Minaee S. Fotouhi M. Khalaj B. H. (2014). “A geometric approach to fully automatic chromosome segmentation,” in 2014 IEEE Signal Processing in Medicine and Biology Symposium (SPMB), 20 July 2015 (Philadelphia, PA, USA: IEEE), 1–6. 10.1109/SPMB.2014.7163174

[B19] Paszke A. Gross S. Massa F. Lerer A. Bradbury J. Chanan G. (2019). “Pytorch: An imperative style, high-performance deep learning library,” in Advances in Neural Information Processing Systems 32: Annual Conference on Neural Information Processing Systems 2019, NeurIPS 2019, Vancouver, BC, Canada, December 8-14, 2019, 8024–8035.

[B20] Szegedy C. Vanhoucke V. Ioffe S. Shlens J. Wojna Z. (2016). “Rethinking the inception architecture for computer vision,” in 2016 IEEE Conference on Computer Vision and Pattern Recognition, CVPR 2016, June 27-30, 2016 (Las Vegas, NV, USA: IEEE Computer Society), 2818–2826. 10.1109/CVPR.2016.308

[B21] van den Oord A. Li Y. Vinyals O. (2018). Representation learning with contrastive predictive coding. CoRR abs/1807.03748.

[B22] Wang F. Cheng J. Liu W. Liu H. (2018a). Additive margin softmax for face verification. IEEE Signal Process. Lett. 25, 926–930. 10.1109/LSP.2018.2822810

[B23] Wang H. Wang Y. Zhou Z. Ji X. Gong D. Zhou J. (2018b). “Cosface: Large margin cosine loss for deep face recognition,” in 2018 IEEE Conference on Computer Vision and Pattern Recognition, CVPR 2018, June 18-22, 2018 (Salt Lake City, UT, USA: Computer Vision Foundation/IEEE Computer Society), 5265–5274. 10.1109/CVPR.2018.00552

[B24] Wu Z. Xiong Y. Yu S. X. Lin D. (2018). “Unsupervised feature learning via non-parametric instance discrimination,” in 2018 IEEE Conference on Computer Vision and Pattern Recognition, CVPR 2018, June 18-22, 2018 (Salt Lake City, UT, USA: Computer Vision Foundation/IEEE Computer Society), 3733–3742. 10.1109/CVPR.2018.00393

[B25] Xie S. Girshick R. B. Dollár P. Tu Z. He K. (2017). “Aggregated residual transformations for deep neural networks,” in 2017 IEEE Conference on Computer Vision and Pattern Recognition, CVPR 2017, July 21-26, 2017 (Honolulu, HI, USA: IEEE Computer Society), 5987–5995. 10.1109/CVPR.2017.634

[B26] Yilmaz I. C. Yang J. Altinsoy E. Zhou L. (2018). “An improved segmentation for raw g-band chromosome images,” in 5th International Conference on Systems and Informatics, ICSAI 2018, November 10-12, 2018 (Nanjing, China: IEEE), 944–950. 10.1109/ICSAI.2018.8599328

[B27] Yun S. Han D. Chun S. Oh S. J. Yoo Y. Choe J. (2019). “Cutmix: Regularization strategy to train strong classifiers with localizable features,” in 2019 IEEE/CVF International Conference on Computer Vision, ICCV, October 27 - November 2, 2019 (Seoul, Korea (South): IEEE), 6022–6031. 10.1109/ICCV.2019.00612

[B28] Zhang H. Cissé M. Dauphin Y. N. Lopez-Paz D. (2018). “mixup: Beyond empirical risk minimization,” in 6th International Conference on Learning Representations, ICLR 2018, April 30 - May 3, 2018 (Vancouver, BC, Canada: OpenReview.net). Conference Track Proceedings.

