# Supplementary material for: SupCAM: Chromosome cluster types identification using supervised contrastive learning with category-variant augmentation and self-margin loss
Source: Front Genet. 2023 Feb 15;14:1109269. doi: 10.3389/fgene.2023.1109269 (PMC9974818; doi:10.3389/fgene.2023.1109269)
Supplement: Supplementary file 1 [file DataSheet1.pdf]

Supplementary Material

1 SUPPLEMENTARY FIGURES

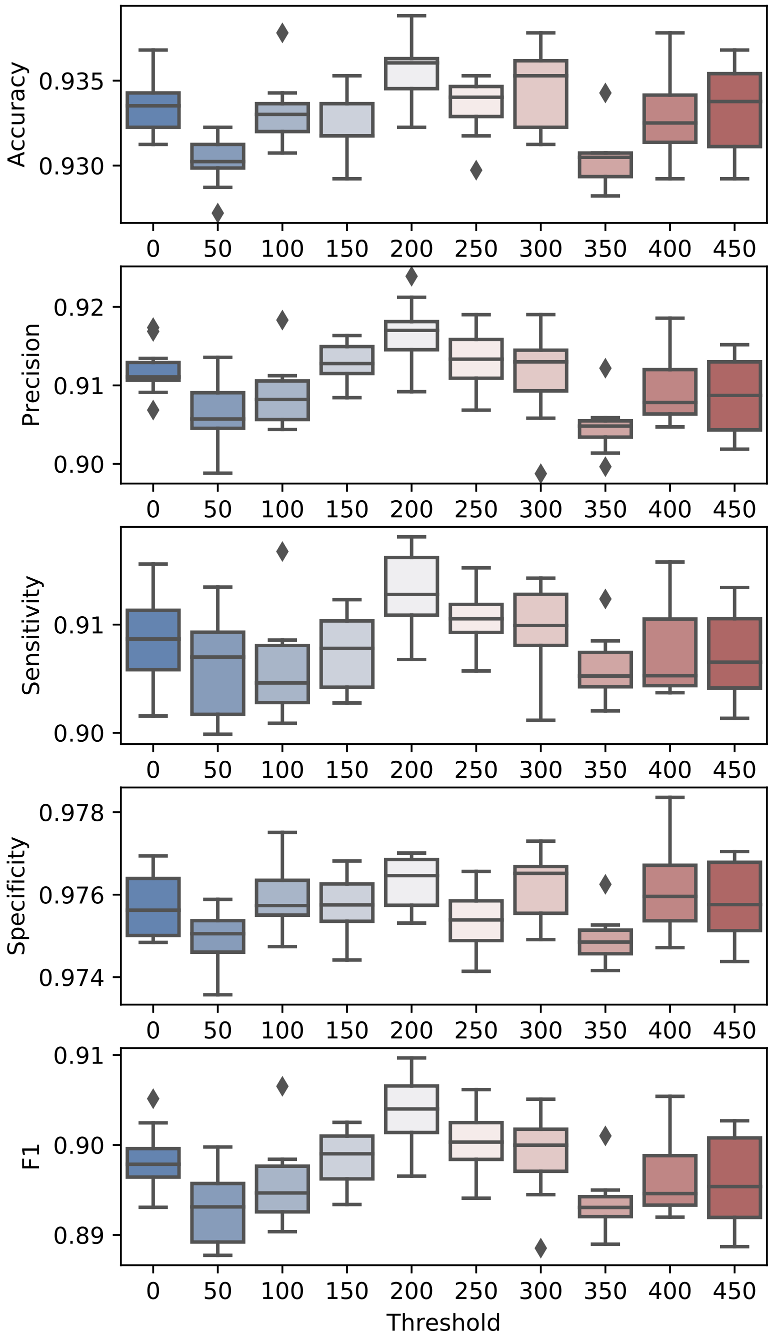

Figure S1. Box plots of 'Accuracy', 'Precision', 'Sensitivity', 'Specificity', and 'F1' under thresholds $P_\cap \in \{0, 50, 100, 150, 200, 250, 300, 350, 400, 450\}$.

2 SUPPLEMENTARY TABLES

Table S1. ImageNet pre-trained SupCAM experiments. SupCAM$^{\natural}$ represents experiments in which we retrained the first step of SupCAM using ImageNet pre-trained weights rather than random initialization.

Methods	Backbone	Pretrain Dataset	Accuracy	Precision	Sensitivity	Specificity	F1
Lin et al.(2021)	ResNet101	ImageNet(1.2M)	91.89	90.65	87.92	97.30	88.32
	DenseNet121	ImageNet(1.2M)	87.65	85.59	81.68	95.88	82.23
	ResNeXt101	ImageNet(1.2M)	92.27	90.79	89.10	97.42	89.36
SupCAM$^{\natural}$	ResNet101	IN+ChrCluster(6.5K)	93.97	91.80	91.62	97.72	90.87
	DenseNet121	IN+ChrCluster(6.5K)	93.85	91.29	91.15	97.62	90.23
	ResNeXt101	IN+ChrCluster(6.5K)	94.08	92.21	91.80	97.73	91.06
SupCAM	ResNet101	ChrCluster(6.5K)	94.24	92.54	92.00	97.74	91.37
	DenseNet121	ChrCluster(6.5K)	94.69	92.92	92.89	97.94	92.11
	ResNeXt101	ChrCluster(6.5K)	**94.99**	**93.25**	**92.81**	**98.12**	**92.26**

As shown in Table S1, we have retrained the first step of SupCAM using ImageNet pre-trained weights as initial weights rather than random initialization, called SupCAM$^{\natural}$. The improvement of SupCAM$^{\natural}$ is derived from introducing ChrCluster which are close to the goal task, thus got domain-friendly weights in the pretraining step. Besides, our final method, SupCAM, achieves best performance among these methods using only the ChrCluster dataset, because all features are derived from medical objects. Without irrelevant prior knowledge (ImageNet pre-trained weights), SupCAM gets easier to pretrain representative and domain-friendly weights for the downstream classification task.
